# Increased Levels of VEGF-A and HIF-1α in Turkish Children with Crimean-Congo Hemorrhagic Fever

**Published:** 2017-03-14

**Authors:** Murat Sefikogullari, Ali Kaya, Huseyin Aydin, Enver Sancakdar, Veysel Kenan Celik, Gokhan Bagci

**Affiliations:** 1 Pediatric Clinic, Private Samandağ Medical Center, Hatay, Turkey; 2 Department of Pediatrics, Faculty of Medicine, Cumhuriyet University, Sivas, Turkey; 3 Department of Biochemistry, Faculty of Medicine, Cumhuriyet University, Sivas, Turkey

**Keywords:** Crimean congo hemorrhagic fever, VEGF, HIF-1α, Sepsis, Children

## Abstract

**Background:**

Crimean-Congo Hemorrhagic Fever (CCHF) is a disease characterized by serious course, including acute viral fever, ecchymosis, thrombocytopenia, liver dysfunction and high rate of mortality. Hypoxia Inducible Factor-1α (HIF-1α) and Vascular Endothelial Growth Factor-A (VEGF-A) play an important role both in the inflammatory process and plasma leakage. The aim of this study was to define HIF-1α and VEGF-A serum levels obtained from CCHF patients and control group and to investigate whether these factors were correlated with the pathogenesis of this disease.

**Methods:**

Thirty cases younger than 17yr confirmed by RT-PCR and/or ELISA for CCHF were included in this study. Thirty age and sex matched healthy peoples were enrolled as controls. Blood samples collected from the patient and control groups. Serum levels of HIF-1α and VEGF-A were measured with ELISA.

**Results:**

Levels of HIF-1α and VEGF-A were statistically significantly increased in CCHF patients compared to the control group (P< 0.05). A significant positive correlation was found between the levels of HIF-1α and VEGF-A in the patient group (P< 0.01). The levels of ALT, AST, CK, aPTT, WBC and Thrombocyte count were significantly higher in the patients than in the control group (P< 0.001). A positive correlation was found among the levels of AST and CK from biochemical parameters and VEGF and HIF-1α in the patient group (P< 0.05)

**Conclusion:**

HIF-1α and VEGF-A might play an important role in CCHF pathogenesis.

## Introduction

Crimean-Congo Hemorrhagic Fever (CCHF) is a serious disease occurring in case of infection by tick-borne virus (*Nairovirus*) genus from Bunyaviridae family (Ergonul 2008). It begins with fever, severe headache, nausea, weakness, vomiting and progresses with bleeding in different parts of the body. “The bleeding findings emerge as subcutaneous bleeding, nasal bleeding, gingival bleeding and visceral bleeding” (Bakir et al. 2005). Mononuclear phagocytic cells, hepatocytes and endothelial cells are known as main target of CCHF virus (Whitehouse 2004, Ergonul et al. 2004).

CCHF cases have been reported from more than 30 countries of Asia, South-Eastern Europe and Africa until now (Hubalek and Rudolf 2012). In Turkey, annually more than 1,000 human CCHF cases are reported (Maltezou et al. 2010).

Like sepsis, endothelial damage plays an important role in the pathogenesis of CCHF. Endothelium may be indirectly targeted by virus mediated host derived soluble factors or directly by viral factors (Schnittler and Feldmann 2003). Hypoxia inducible factor (HIF-1α) is an oxygen sensitive transcription factor facilitating oxygen distribution and cellular adaptation to oxygen deprivation. HIF-1α activates transcription of genes, which encode angiogenic growth factors, including vascular endothelial growth factor (VEGF), angiopoietin 1 (ANGPT1), (ANGPT2), placental growth factor (PGF) and platelet-derived growth factor B (PDGFB), playing a critical role in angiogenesis. HIF-1α regulates expression of certain genes related with erythropoiesis, energy metabolism, angiogenesis, cell proliferation and apoptosis (Ferrara et al. 2003, Podar and Anderson 2005, Manalo et al. 2005).

Vascular Endothelial Growth Factor (VEGF-A), a member of platelet-derived growth factors super family is specific for the endothelial cells and has important effects. Blood vessel formation is regulated mainly by vascular endothelial growth factor (VEGF-A) in health and disease. Human VEGF gene family consists of five members of VEGF A-E, among which VEGF-A is also commonly referred to as VEGF (Ferrara 2003).

VEGF-A was also known as vascular permeability factor (VPF) due to its substantial effect on increasing of vascular permeability, resulting in a leakage out of blood vessels. One of the main aberrations in pathogenesis of CCHF is vascular dysfunction resulting in leakage driven hemorrhagic manifestations (Dvorak et al. 1995, Hoeben et al. 2004, Ergonul 2006).

In this study, we aimed to demonstrate whether VEGF-A and HIF-1α was correlated with pathogenesis of sepsis in serum samples obtained from CCHF patients and control group.

## Materials and Methods

### Study design

Thirty patients diagnosed with CCHF in Department of Pediatrics, Faculty of Medicine, Cumhuriyet University, from 2010 to 2011 and 30 healthy controls were enrolled in the study. All of the patients with CCHF used to live in the same endemic region (Sivas and neighbor’s cities) which showed high prevalence in CCHF. Blood samples collected from the hospitalized patients with presumed diagnosis of CCHF were sent to Refik Saydam Hifzisihha Center, Virology Laboratory. Thirty patients having positive CCHFV RNA with Reverse Transcriptase-Polymerase Chain Reaction (RT-PCR) and/or positive CCHFV-specific IgM with ELISA were included in the study. Thirty age and sex matched healthy individuals who had not any infectious or metabolic diseases were enrolled as the controls.

This study was approved by the Local Ethics Committee of Cumhuriyet University, Faculty of Medicine.

### Measurement of HIF-1α and VEGF-A levels

Blood samples of 5 ml were collected from patient and control groups. The samples were centrifuged at 3000 rpm for 5 min and obtained serums were portioned and kept at −80 °C until the analysis time. Then these samples were cooled to the room temperature at the same time and HIF-1α and VEGF-A levels were measured through ELISA method. Human/Mouse Total HIF-1 alpha Cell-Based ELISA (R and D Systems, Inc.) and VEGF-A Human (BioVendor) kits were used to measure the levels of HIF-1α and VEGF-A. MicroELISA (sandwich) method was performed using full automatic TRITURUS device.

### Statistical analysis

All statistical analyses were conducted with IBM SPSS Statistics for Windows, Version 20.0 (Chicago, IL, USA) computer program. Descriptive statistics were expressed as arithmetic mean (min-max). Mann–Whitney U test was performed to determine the significance of independent continuous variables. For bivariate correlation analysis, Pearson’s or Spearman’s test was used. In evaluation of the categorical data, Chi-square test was used in the statistical analyses. P< 0.05 values were considered as statistically significant for all the tests.

## Results

Demographic features and laboratory findings of the controls and patient groups are given in Table 1. Mean age was found as 11.30±4.41 in the patients and 10.40±4.49 in the controls. Nineteen of the patients were male (63.3%) and 11 were female (36.7%), whereas 20 (66.7%) of the controls were male and 10 (33.3%) were female. The differences between the controls and patient groups in terms of age and gender were not significant.

**Table 1.** Comparison of demographic and laboratory findings of the patients and controls

**Variable**	**Control (n=30)**	**CCHF (n=30**)	**P**
**Age (mean±SD)**	10.40±4.49	11.30±4.41	0.05
**Age range**	2–17	2–17	0.05
**Male (%)**	20 (66.7%)	19 (63.3%)	0.05
**Female (%)**	20 (66.7%)	19 (63.3%)	0.05
**ALT (IU/L)**	35 (12–48)	83 (28–335)	**<0.001**
**AST (IU/L)**	38 (11–54)	171 (26–357)	**<0.001**
**CK (IU/L)**	107 (68–172)	899 (140–1235)	**<0.001**
**Prothrombin time (s)**	14.4 (9.6–14.0)	15.9 (9.6–20.1)	>0.05
**aPTT (s)**	34.7 (13.5–45.7)	42.4 (23.8–55.1)	**0.022**
**INR**	1.2 (0.8–2.2)	1.3 (0.9–2.4)	>0.05
**WBC (×10^9^ cells/L)**	5.7 (3.5–7.0)	3.17 (1.2–9.0)	**<0.05**
**Hemoglobin (g/dL)**	14.2 (8.3–15.8)	13.6 (11.8–15.8)	>0.05
**Thrombocyte count (×10^3^ cells/L)**	176 (69–214)	110 (38–152)	**<0.001**

**ALT:** Alanine aminotransferase

**AST:** Aspartate aminotransferase

**CK:** Creatine kinase

**aPTT:** activated partial thromboplastin time

**INR:** International normalized ratio

**WBC:** White blood cell

A statistically significant increase was observed in the levels of HIF-1α and VEGF in the patients compared to control groups (P< 0.05, Table 2). A significant positive correlation was found between the levels of HIF-1α and VEGF-A in patient group (P< 0.01, Table 2). A positive correlation was found among the levels of AST and CK from biochemical parameters and VEGF and HIF-1α (P< 0.05), while this was not significant in ALT (P= 0.086, Table 3).

**Table 2.** Serum levels of VEGF-A and HIF-1α in the patient and control groups

	**Patients**	**Control**	**P**
**VEGF-A (pg/ml)**	312.66±87.31	150.70±78.48	**0.001**
**HIF-1α (pg/ml)**	195.93±63.49	68.20±36.11	**0.001**

**Table 3.** HIF-1α and VEGF-A correlation between biochemical parameters in patients with CCHF

**Variable**	**HIF-1α**	**VEGF-A**		

	**Correlation Coefficient**	**P**	**Correlation Coefficient**	**P**
**ALT**	0.181	0.086	0.162	0.103
**AST**	0.256	**0.020**	0.266	**0.022**
**CK**	0.214	**0.012**	0.292	**0.048**
**VEGF-A**	0.969	**<0.01**	-	-

**ALT:** Alanine aminotransferase

**AST**: Aspartate aminotransferase

**CK**: Creatine kinase

The levels of ALT, AST and CK were statistically significantly higher in CCHF patients than in the control group (P< 0.001). There was a significant increase in the levels of aPTT in coagulation tests compared to controls (P=0.022), increase in the prothrombin time and INR was not statistically significant (P> 0.05 Table 1). WBC (P< 0.05) and thrombocyte counts (P< 0.001) were statistically lower in the patients than in the control group, while no significant difference was observed in the amount of hemoglobin.

## Discussion

We aimed to demonstrate whether HIF-1α and VEGF, related to sepsis and viral infection, were correlated with pathogenesis of CCHF disease.

Despite numerous studies conducted about CCHF, pathogenesis of the disease is not fully understood. There was a relationship between sepsis and viral infection, and HIF-1α and VEGF (Irwin et al. 2009). In infected humans by the CCHFV, damaging of endothelial cells and vascular leakage may be a direct result of an immune mediated indirect effect (Schnittler and Feldmann 2003).

A common characteristic of viral hemorrhagic fevers is viruses to enter macrophages and dendritic cells, making a cytopathic effect. VEGF produced by monocytes and endothelial cells is known to play an important role in breakdown of coagulation, leukocyte adhesion, angiogenesis and increased vascular permeability (Burt et al. 1997, Geisbert and Jahrling 2004). It is known as a deteriorated coagulation, leukocyte adhesion, angiogenesis and increased vascular permeability in CCHF patients (Bodur et al. 2010).

Clinical symptoms of CCHF are similar to sepsis and endothelial structure of vessels is retrograded (Elson et al. 2001). The correlation of sepsis and viral infection with HIF-1α and VEGF-A was shown (Kilani et al. 2004, Zinkernagel et al. 2007).

At hypoxic conditions, hypoxia-inducible factor (HIF) regulates expression of certain genes. In *in vivo* and *in vitro* studies, oxygen tension affects virus production either up regulating or down regulating viral replication depending on virus type and method used for to study viral replication. Viral replication is modulated mainly by HIF-1α in normal and hypoxic conditions (Morinet et al. 2013).

HIF-1α is known to activate transcription of the genes including VEGF and other angiogenic growth factors and plays a critical role in the development of angiogenesis in hypoxic conditions (Manalo et al. 2005). HIF-1α mRNA expression was suppressed and conversely associated with disease severity in sepsis (Schaefer et al. 2013). HIF-1α expression was statistically significantly higher in the shock patients compared to the controls and the reasons of shock were sepsis (78%), hemorrhage (18%), and cardiac dysfunction (4%) (Textoris et al. 2012).

In sepsis, microvascular permeability closely associates with the increased plasma VEGF concentration (Pickkers et al. 2005). ROS–HIF-1α–VEGF pathway is partly responsible for hypoxia-induced permeability (Irwin et al. 2009). The levels of VEGF increased in sepsis, and this increase correlated with severity of the disease and mortality (van der Flier et al. 2005, Yano et al. 2006, Karlsson et al. 2008).

Inhibition of VEGF signaling contributes the development of sepsis-induced organ dysfunction via blocking endothelial survival and increasing apoptosis. Furthermore, in pathological situations like sepsis and cancer, VEGF participates to mobilizing of endothelial progenitor cells (Jesmin et al. 2012).

The main target of sepsis-induced events is generally endothelium, so ability to ameliorate damaged endothelium is one of the main determiners of clinical outcome in septic patients. The angiogenic factors and their soluble receptors have a dual role and seem to play both beneficial and harmful effects during sepsis development and therapy. While normal levels of VEGF is required for protection of endothelial function, both extremely high or low levels of VEGF have disruptive effect on endothelial barrier (Zhang et al. 2013).

In our study, a strong correlation was seen between the levels of HIF-1α and VEGF-A in CCHF patients (Table 3). Besides, a positive correlation was found between HIF-1α and VEGF-A, suggesting that angiogenesis could be triggered and, pathogenesis of the disease could be influenced. Accordingly, we recommend that comprehensive studies related to angiogenesis should be conducted in CCHF patients.

Despite the absence of studies that investigated HIF-1α levels in patients with CCHF in the literature, the studies that evaluating VEGF levels have increased in recent years. All of the studies that investigate serum levels of VEGF have been conducted with Turkish CCHF patients. Serum levels of VEGF was statistically higher in the adult patients having CCHF compared to the controls (Bakir et al. 2013), and in another study, levels of VEGF was found statistically higher in fatal CCHF patients compared to the non-fatal patients (Ozturk et al. 2010). In contrast to these findings, level of VEGF was significantly decreased in CCHF patients (Bodur et al. 2010). Our study was not consistent with the study by Bodur et al. while it was consistent with the other two studies.

In studies conducted with other hemorrhagic fevers such as dengue hemorrhagic fever (DHF) (Tseng et al. 2000, Srikiatkhachorn et al. 2007, Furuta et al. 2012, del Moral-Hernández et al. 2014) and hemorrhagic fever with renal syndrome (HFRS) (Ma et al. 2012), also VEGF level was statistically significantly higher in patient groups. Consequently, all of these studies indicate that VEGF regulates vascular permeability and VEGF is a good marker of the severity of the infection by viral diseases such as CCHF and DHF.

Our study has some limitations. First, we could not linked the levels of HIF-1α and VEGF-A together with disease severity. Second, HIF-1α and VEGF-A serum levels were not compared between patients with survival and non-survival. Lastly, we did not evaluate the levels of VEGF-A and HIF-1α in another viral disease -especially the other hemorrhagic fevers- or patients with sepsis.

Increased levels of the HIF-1α which is the main transcriptional factor of the regulation of oxygen homeostasis and VEGF-A which has a crucial role in the regulation of angiogenesis and vascular permeability suggested that these parameters might contribute to the development of vascular endothelial damage. Nevertheless, because of the insufficient information about molecular mechanism on pathogenesis of CCHF, our results indicate that VEGF and HIF-1α should be one of the markers need to be focused on the elucidation on the pathogenesis of CCHF disease.

## Conclusion

Significant higher levels of HIF-1α and VEGF-A in the CCHF patients than in controls, suggested that HIF-1α and VEGF-A expression might be effective on the pathogenesis of CCHF patients, especially on the occurrence of endothelial damage and disruption of coagulation. Combined use of HIF-1α inhibitors with antiviral agents may be useful for the treatment of CCHF patients. Further comprehensive studies on this issue are recommended.

## References

[B1] Bakir M Ugurlu M Dokuzoguz B Bodur H Tasyaran MA Vahaboglu H ( 2005) Turkish CCHF Study Group. Crimean-Congo haemorrhagic fever outbreak in Middle Anatolia: a multicentre study of clinical features and outcome measures. J Med Microbiol. 54: 385– 389. 15770025 10.1099/jmm.0.45865-0

[B2] Bakir M Bakir S Sari I Celik VK Gozel MG Engin A ( 2013) Evaluation of the relationship between serum levels of VEGF and sVEGFR1 with mortality and prognosis in patients with Crimean-Congo hemorrhagic fever. J Med Virol. 85: 1794– 1801. 23868816 10.1002/jmv.23666

[B3] Bodur H Akinci E Onguru P Uyar Y Basturk B Gozel MG Kayaslan BU ( 2010) Evidence of vascular endothelial damage in Crimean-Congo hemorrhagic fever. Int J Infect Dis. 14: e704– e707. 20627646 10.1016/j.ijid.2010.02.2240

[B4] Burt FJ Swanepoel R Shieh WJ Smith JF Leman PA Greer PW Coffield LM Rollin PE Ksiazek TG Peters CJ Zaki SR ( 1997) Immunohistochemical and in situ localization of Crimean-Congo hemorrhagic fever (CCHF) virus in human tissues and implications for CCHF pathogenesis. Arch Pathol Lab Med. 121: 839– 846. 9278612

[B5] Del Moral-Hernández O Martínez-Hernández NE Mosso-Pani MA Hernández-Sotelo D Illades-Aguiar B Flores-Alfaro E Antonio-Vejar V Leyva-Vázquez MA ( 2014) Association DENV1 and DENV2 infection with high serum levels of soluble thrombomodulin and VEGF in patients with dengue fever and dengue hemorrhagic fever. Int J Clin Exp Med. 7: 370– 378. 24600491 PMC3931590

[B6] Dvorak HF Brown LF Detmar M Dvorak AM ( 1995) Vascular permeability factor/vascular endothelial growth factor, microvascular hyperpermeability, and angiogenesis. Am J Pathol. 146: 1029– 1039. 7538264 PMC1869291

[B7] Elson DA Thurston G Huang LE Ginzinger DG McDonald DM Johnson RS Arbeit JM. ( 2001) Induction of hypervascularity without leakage or inflammation in transgenic mice overexpressing hypoxia-inducible factor-1α. Genes Dev. 15: 2520– 2532. 11581158 10.1101/gad.914801 PMC312791

[B8] Ergönül O Celikbas A Dokuzoguz B Eren S Baykam N Esener H ( 2004) Characteristics of patients with Crimean-Congo Hemorrhagic Fever in a recent outbreak in Turkey and impact of oral ribavirin therapy. Clin Infect Dis. 39: 284– 287. 15307042 10.1086/422000

[B9] Ergonul O ( 2006) Crimean-Congo haemorrhagic fever. Lancet Infect Dis. 6: 203– 214. 16554245 10.1016/S1473-3099(06)70435-2 PMC7185836

[B10] Ergonul O ( 2008) Treatment of Crimean-Congo hemorrhagic fever. Antiviral Res. 78: 125– 131. 18096251 10.1016/j.antiviral.2007.11.002

[B11] Ferrara N Gerber HP Lecouter J ( 2003) The biology of VEGF and its receptors. Nat Med. 9: 669– 676. 12778165 10.1038/nm0603-669

[B12] Furuta T Murao LA Lan NTP Huy NT Huong VTQ Huy TT Tham VD Nga CTP Ha TTN Ohmoto Y Kikuchi M Morita K Yasunami M Hirayama K Watanabe N ( 2012) Association of mast cell-derived VEGF and proteases in dengue shock syndrome. PLOS Negl Trop Dis. 6: e1505. 22363824 10.1371/journal.pntd.0001505 PMC3283553

[B13] Geisbert TW Jahrling PB ( 2004) Exotic emerging viral diseases: progress and challenges. Nat Med. 10 (12 Suppl): S110– S121. 15577929 10.1038/nm1142

[B14] Hoeben A Landuyt B Highley MS Wildiers H Van Oosterom AT De Bruijn EA ( 2004) Vascular endothelial growth factor and angiogenesis. Pharmacol Rev. 56: 549– 580. 15602010 10.1124/pr.56.4.3

[B15] Hubalek Z Rudolf I ( 2012) Tick-borne viruses in Europe. Parasitol Res. 111: 9– 36. 22526290 10.1007/s00436-012-2910-1

[B16] Irwin DC McCord JM Nozik-Grayck E Beckly G Foreman B Sullivan T White M Crossno Jr JT Bailey D Flores SC Majka S Klemm D Martha C TissotvanPatot MC ( 2009) A potential role for reactive oxygen species and the HIF-1α–VEGF pathway in hypoxia-induced pulmonary vascular leak. Free Radic Biol Med. 47: 55– 61. 19358884 10.1016/j.freeradbiomed.2009.03.027 PMC2689923

[B17] Jesmin S Zaedi S Islam AS Sultana SN Iwashima Y Yamaguchi N Hiroe M Gando S ( 2012) Time-dependent alterations of VEGF and its signaling molecules in acute lung injury in a rat model of sepsis. Inflammation. 35: 484– 500. 21528367 10.1007/s10753-011-9337-1

[B18] Karlsson S Pettilä V Tenhunen J Lund V Hovilehto S Ruokonen E Finnsepsis Study Group ( 2008) Vascular endothelial growth factor in severe sepsis and septic shock. Anesth Analg. 106: 1820– 1826. 18499616 10.1213/ane.0b013e31816a643f

[B19] Kilani MM Mohammed KA Nasreen N Tepper RS Antony VB ( 2004) RSV causes HIF-1 alpha stabilization via NO release in primary bronchial epithelial cells. Inflammation. 28: 245– 251. 16133997 10.1007/s10753-004-6047-y

[B20] Ma Y Liu B Yuan B Wang J Yu H Zhang Y Xu Z Zhang Y Yi J Zhang C Zhou X Yang A Zhuang R Jin B ( 2012) Sustained high level of serum VEGF at convalescent stage contributes to the renal recovery after HTNV infection in patients with hemorrhagic fever with renal syndrome. Clin Dev Immunol. 2012: 812386. 23097674 10.1155/2012/812386 PMC3477746

[B21] Maltezou HC Andonova L Andraghetti R Bouloy M Ergonul O Jongejan F Kalvatchev N Nichol S Niedrig M Platonov A Thomson G Leitmeyer K Zeller H ( 2010) Crimean-Congo hemorrhagic fever in Europe: current situation calls for preparedness. Euro Surveill. 11; 15( 10): 19504. 20403306

[B22] Manalo DJ Rowan A Lavoie T Natarajan L Kelly BD Ye SQ Garcia JGN Semenza GL ( 2005) Transcriptional regulation of vascular endothelial cell responses to hypoxia by HIF-1. Blood. 105: 659– 669. 15374877 10.1182/blood-2004-07-2958

[B23] Morinet F Casetti L François JH Capron C Pillet S ( 2013) Oxygen tension level and human viral infections. Virology. 444: 31– 36 23850460 10.1016/j.virol.2013.06.018

[B24] Ozturk B Kuscu F Tutuncu E Sencan I Gurbuz Y Tuzun H ( 2010) Evaluation of the association of serum levels of hyaluronic acid, sICAM-1, sVCAM-1, and VEGF-A with mortality and prognosis in patients with Crimean-Congo hemorrhagic fever. J Clin Virol. 47: 115– 119 20005156 10.1016/j.jcv.2009.10.015

[B25] Pickkers P Sprong T van Eijk L van der Hoeven H Smits P van Deuren M ( 2005) Vascular endothelial growth factor is increased during the first 48 hours of human septic shock and correlates with vascular permeability. Shock. 24: 508– 512. 16317379 10.1097/01.shk.0000190827.36406.6e

[B26] Podar K Anderson KC ( 2005) The pathophysiologic role of VEGF in hematologic malignancies therapeutic implications. Review, Blood. 105: 1383– 1395. 15471951 10.1182/blood-2004-07-2909

[B27] Schnittler HJ Feldmann H ( 2003) Viral hemorrhagic fever a vascular disease? Thromb Haemost. 89: 967– 972. 12783108

[B28] Schäfer ST Frede S Winning S Bick A Roshangar P Fandrey J Peters J Adamzik M ( 2013) Hypoxia-inducible factor and target gene expression are decreased in patients with sepsis: prospective observational clinical and cellular studies. Anesthesiology 118: 1426– 1436. 23449494 10.1097/ALN.0b013e31828baa67

[B29] Srikiatkhachorn A Ajariyakhajorn C Endy TP Kalayanarooj S Libraty DH Green S Ennis FA Rothman AL ( 2007) Virus-induced decline in soluble vascular endothelial growth receptor 2 is associated with plasma leakage in dengue hemorrhagic Fever. J Virol. 81: 1592– 1600. 17151115 10.1128/JVI.01642-06 PMC1797579

[B30] Textoris J Beaufils N Quintana G Lassoued AB Zieleskiewicz L Wiramus S Blasco V Lesavre N Martin C Gabert J Leone M ( 2012) Hypoxia-inducible factor (HIF1α) gene expression in human shock states. Crit Care. 16( 4): R120. doi: 10.1186/cc11414. 22781303 PMC3580697

[B31] Tseng CS Lo HW Teng HC Lo WC Ker CG ( 2005) Elevated levels of plasma VEGF in patients with dengue hemorrhagic fever. FEMS Immunol Med Microbiol. 43: 99– 102. 15607642 10.1016/j.femsim.2004.10.004

[B32] Van der Flier M van Leeuwen HJ van Kessel KP Kimpen JL Hoepelman AI Geelen SP ( 2005) Plasma vascular endothelial growth factor in severe sepsis. Shock. 23: 35– 38. 15614129 10.1097/01.shk.0000150728.91155.41

[B33] Whitehouse CA ( 2004) Crimean-Congo hemorrhagic fever. Antiviral Res 64: 145– 160. 15550268 10.1016/j.antiviral.2004.08.001

[B34] Yano K Liaw PC Mullington JM Shih SC Okada H Boydak N Kang PM Toltl L Belikoff B Buras J Simms BT Mizgerd JP Carmeliet P Karumanchi SA Aird WC ( 2006) Vascular endothelial growth factor is an important determinant of sepsis morbidity and mortality. J Exp Med. 203: 1447– 1458. 16702604 10.1084/jem.20060375 PMC2118329

[B35] Zhang RY Liu YY Qu HP Tang YQ ( 2013) The angiogenic factors and their soluble receptors in sepsis: friend, foe, or both? Crit Care. 17: 446. 23968539 10.1186/cc12857 PMC4056530

[B36] Zinkernagel AS Johnson RS Nizet V ( 2007) Hypoxia inducible factor (HIF) function in innate immunity and infection. J Mol Med. 85: 1339– 1346. 18030436 10.1007/s00109-007-0282-2

